# Comprehensive methylome and transcriptome profiling reveals specific biomarkers for bovine viral diarrhea virus persistent infection in calves

**DOI:** 10.3389/fimmu.2026.1763258

**Published:** 2026-03-10

**Authors:** Jiahao Wang, Siqian Chen, Wanyi Lai, Xiao Feng, Qingyao Zhao, Siyuan Mi, Chuang Xu, Tong Qin, Jie Cao, Ying Yu

**Affiliations:** 1 National Engineering Laboratory for Animal Breeding, State Key Laboratory of Animal Biotech Breeding, Breeding and Reproduction of Ministry of Agriculture and Rural Affairs, College of Animal Science and Technology, China Agricultural University, Beijing, China; 2 College of Veterinary Medicine, China Agricultural University, Beijing, China; 3 Key Laboratory of Animal Biosafety Risk Prevention and Control (North) & Key Laboratory of Veterinary Biological Products and Chemical Drugs of MARA, Institute of Animal Science, Chinese Academy of Agricultural Sciences, Beijing, China

**Keywords:** biomarker, bovine viral diarrhea virus, DNA methylation, persistently infected, trojan dam

## Abstract

**Background:**

Persistently infected (PI) calves resulting from maternal Bovine viral diarrhea virus (BVDV) infection during early gestation are the main source of viral transmission and pose a serious threat to the sustainable production of herds. PI cattle appear clinically normal, elucidating the molecular-level alterations is critical for understanding their specific characteristics. Moreover, the identification of candidate biomarkers for diagnosing PI cattle will provide valuable insights to support effective strategies for the control and eventual eradication of BVDV. At the same time, few studies have focused on the mothers of PI calves (Trojan dam).

**Methods:**

We integrated RNA-seq and reduced representation bisulfite sequencing (RRBS) to characterize the molecular features of PI calves and Trojan dams and identified DNA methylation biomarkers in PI calves.

**Results:**

In this study, we found that both B cell and T cell receptor signaling pathways were less active in PI calves, along with a significantly reduced B cell proportion compared to normal calves. By integrating transcriptome and methylation data, we found that the elevated expression of *MAPK13* and *LSP1* in PI calves is potentially regulated by promoter methylation. Furthermore, we identified a potential ZBTB38–methylation–MAPK13 regulatory axis influencing downstream genes such as *IL1B*, *KDR*, *FOSB*, and *PTGS2*, which may collectively impact the physiological state of PI calves. In Trojan dams and normal dams, we identified a total of eight genes potentially regulated by DNA methylation, including *KLHL33*, *KLRG1*, *NQO2* and etc. By comparing PI calves with Trojan dams, normal calves, and normal dams, we identified a differentially methylated region (DMR) located in the *PES1* gene that was specifically hypomethylated in PI calves but hypermethylated in the other groups, suggesting its potential as a candidate diagnostic biomarker for PI detection.

**Conclusion:**

Our study revealed that the immune system of PI cattle is already compromised and identified a novel candidate diagnostic biomarker, providing a new perspective for the detection of PI cattle. In addition, we reveal that prenatal BVDV exposure induces persistent DNA methylation alterations in Trojan dams.

## 1 Introduction

Bovine viral diarrhea virus (BVDV) is a single-stranded, positive-sense RNA virus that belongs to the genus *Pestivirus* in the family *Flaviviridae* (1, 2). It is associated with gastrointestinal, respiratory, and reproductive diseases, and is widely distributed around the world, causing a significant economic burden on the global livestock industry (3–5). Based on the ability to cause cytopathy in cultured cells *in vitro*, BVDV can be classified into cytopathic and noncytopathic types (6). Fetuses infected with the noncytopathic strain of BVDV between days 45 and 125 of gestation develop into persistently infected (PI) calves after birth (7, 8). PI animals are clinically healthy, and while some individuals can survive into adulthood, they continuously shed BVDV throughout their lives, acting as the primary source of infection and causing substantial economic losses for farms (9, 10). Knapek et al. (11) compared immune-related genes between PI cattle and normal cattle and found significant downregulation of genes involved in innate immunity (*NFKB*, *IFNB*) and adaptive immunity (*PSMB9*, *TAP1*, *B2M*, *CD4*), indicating that BVDV infection leads to a decline in immune function in PI cattle. Exploring the molecular regulatory network of PI cattle will deepen our understanding of the mechanisms underlying PI formation.

The development of PI cattle occurs when a fetus is infected with BVDV during a period before immunocompetence is fully established, regardless of the animal’s genetic background (8, 10, 12). The epigenome describes heritable structural modifications of the genome that regulate the expression of genetic information, including DNA methylation and histone modifications (13, 14). Meanwhile, epigenetic modifications are highly plastic and susceptible to modulation by viruses and other environmental factors (15, 16). Human influenza virus infection induces alterations in epigenetic regulation of inflammation-related genes, such as decreased expression of *MAPK13* in the JNK cascade, hypermethylation of its promoter region, and reduced levels of the histone acetylation mark H3K27ac (17). Santarelli et al. (18) investigated whole-genome DNA methylation in EBV-infected and uninfected control HCoEpC cells and identified a total of 1,606 differentially methylated regions affecting genes involved in carcinogenesis and inflammation. DNA methylation changes induced by Japanese encephalitis virus in mouse models have been shown to modulate tight junction signaling, which may alter blood-brain barrier permeability (19). Georges et al. (20) analyzed the DNA methylation in the spleens of PI fetuses and normal fetuses, identifying 2,641 differentially methylated regions (DMRs) that affect the immune, skeletal, and nervous systems in PI cattle. This suggests that epigenetic changes induced by BVDV infection play a significant role in fetal development. However, the impact of these altered DNA methylation modifications on gene expression has not been further investigated in the existing studies. At the same time, existing research has mainly focused on PI cattle, with little attention given to the changes occurring in dams of PI calves (Trojan dams).

In this study, we collected 4 healthy dam-calf and 5 Trojan dam-PI calf pairs for genome, transcriptome, and methylome sequencing. We used genomic data for parentage testing to validate pedigree records, while simultaneously assessing potential integration of BVDV into the host genome. Through transcriptome analysis, we explored molecular-level differences between PI calves, Trojan cattle, and normal cattle. By integrating methylation data, we further identified methylation features potentially regulating gene expression changes in PI and Trojan cattle. Finally, we compared the methylation data of PI calves with those of Trojan dams, normal calves, and normal dams to identify PI calf-specific methylation markers. The objective of this study was to enhance our understanding of the health status of PI cattle and to provide novel insights that may facilitate improved diagnostic strategies.

## 2 Materials and methods

### 2.1 Sample collection and diagnosis

A total of 18 Holstein cattle were included in this study, comprising four healthy dam-calf pairs and five Trojan dam-PI calf pairs; all calves were female. All dams and their calves were sourced from the same farm, were not subjected to any experimental viral challenge, and were managed under routine farm production practices prior to calving. All studies were conducted after the calves were born, and BVDV testing was conducted as part of the farm’s routine prevention and control program. Immediately after birth (0 days), newborn calves underwent health status assessment using the BVDV Antigen Test Kit/Serum Plus (IDEXX Laboratories, Westbrook, ME, USA), following the manufacturer’s protocol. Briefly, ear notch tissue plugs (2–3 mm in diameter) were immersed in 250 µL of IDEXX Ear Notch Soaking Buffer and incubated for 12–24 hours at 18-26°C. Subsequently, 50 µL of Detection Solution was added to all wells. Negative and positive controls (50 µL each) and 50 µL of soaking buffer for samples were dispensed into the appropriate wells. Plates were gently mixed and incubated at 37°C for 2 hours, followed by five washes with 300 µL of Wash Solution per well. Next, 100 µL of Conjugate was added and incubated at 18-26°C for 30 minutes, followed by another wash step. Then, 100 µL of TMB Substrate N.12 was added and incubated for 10 minutes at 18-26°C. The reaction was stopped with 100 µL of Stop Solution N.3, and absorbance was measured at 450 nm. Samples with corrected OD values (S – N) > 0.300 were considered positive, those < 0.200 negative, and values between 0.200 and 0.300 were retested using an additional 50 µL of the original soaking buffer.

If the calf was negative, the dam was considered negative and was not tested. For antigen-positive calves, their dams were subsequently tested and all proved negative. To confirm persistent infection (PI), calves that initially tested positive were retested after two weeks. Calves positive on both tests were classified as PI, and their dams were classified as Trojan dams.

Blood samples were collected from the tail vein of dams and the jugular vein of calves into EDTA tubes for DNA and RNA extraction (stored at 4°C) and into serum separator tubes for serum collection.

### 2.2 DNA ,RNA extraction

We used the TIANamp Blood DNA Kit and followed the manufacturer’s instructions to extract DNA from blood samples. For RNA extraction, the buffy coat (white blood cells) was first collected by centrifuging fresh anticoagulated blood at 3,000 rpm (1,107 × g; rotor A12-10P) for 15 minutes. Subsequently, RNA was isolated from whole blood leukocytes using TRIzol reagent (Invitrogen, Carlsbad, CA, USA) following the manufacturer’s instructions. Briefly, white blood cells were lysed with 1 mL of TRIzol reagent and homogenized by pipetting up and down. A 0.25 mL aliquot of the lysate was transferred to a new tube, mixed with an additional 1 mL of TRIzol reagent, and incubated for 5 minutes to allow complete dissociation of nucleoprotein complexes. Chloroform (0.2 mL) was added, followed by incubation for 2–3 minutes and centrifugation at 12,000 × g for 15 minutes at 4°C. The aqueous phase was transferred to a new tube, mixed with 0.5 mL of isopropanol, and incubated for 10 minutes at 4°C, followed by centrifugation at 12,000 × g for 10 minutes. The supernatant was discarded with a micropipettor, and the RNA pellet was washed with 75% ethanol (1 mL), briefly vortexed, centrifuged at 7,500 × g for 5 minutes at 4°C, air-dried for 5–10 minutes, and finally resuspended in 50 µL of RNase-free water.

### 2.3 Serum separation and RT-PCR

Serum separator tubes containing blood samples were allowed to clot at room temperature for approximately 1 h. Following centrifugation at 3,000 rpm (1,107 × g; rotor A12-10P) for 10 minutes at room temperature, the upper serum layer was carefully pipetted and aliquoted into 2.0mL centrifuge tubes for storage at -80°C. RNA was extracted using Viral RNA Extraction Kit (Aidlab Biotechnologies Co., Ltd., Beijing, China) according to the manufacturer’s protocol. First-strand cDNA was synthesized from total RNA using the FastKing gDNA Dispelling RT SuperMix (Tiangen Biotech, Beijing, China) to eliminate genomic DNA contamination, following the manufacturer’s instructions, followed by PCR amplification. Agarose gel electrophoresis was performed for all samples together with DNA markers and both positive and negative controls to verify the presence of viral DNA.

### 2.4 Resequencing and quality control for raw data

We used a Qubit Fluorometer to measure DNA concentration and performed agarose gel electrophoresis to assess DNA integrity, ensuring the quality of DNA for library construction. Library preparation was performed using the MGIEasy Universal DNA Library Prep Set (BGI-Shenzhen, China) following the manufacturer’s recommendations, with index codes added to attribute sequences to each sample. The libraries were sequenced on the DNBSEQ-T7 platform (BGI-Shenzhen, China), generating 150 bp paired-end reads. Using the SOAPnuke v2.1.9 (21), we remove adapters and filter out low-quality bases/reads.

### 2.5 Parentage inference

All clean reads were aligned to the bovine reference genome (ARS-UCD1.2; Ensembl release 110) using BWA v0.7.17 (BWA-MEM algorithm, default parameters) (22), and the alignment results were sorted and converted to BAM files using samtools v1.21 (23). We followed the official GATK Best Practices Workflows to process the BAM files and generate the VCF files of SNPs. In brief, we pre-processed the BAM files using MarkDuplicatesSpark, BaseRecalibrator, and ApplyBQSR to mark duplicate reads and correct systematic errors in base quality scores. Next, we employed HaplotypeCaller (in GVCF mode) to call variants for each sample, generating intermediate GVCF files. These files were then consolidated using GenomicsDBImport. Finally, we performed joint genotyping of all GVCF files using GenotypeGVCFs to produce the VCF file. The SNP variants were filtered using the following criteria: QD < 2.0, MQ < 40.0, FS > 60.0, SOR > 3.0, MQRankSum < -12.5, ReadPosRankSum < -8.0.

We used PLINK v1.90b6.21 (24) to calculate the identity by descent (IBD) between individuals using autosomal biallelic markers (9,226,734). We further used VCFtools v0.1.16 (25) to extract autosomal biallelic markers at 10 kb intervals, and then employed EasyPC (26) to infer parentage between mother-daughter pairs using these markers.

### 2.6 Identification of virus insertion events

Virus insertion events in the host genome were identified following the methodology described by Zhang et al. (27). Briefly, resequencing clean reads were aligned to a combined reference comprising the bovine genome (ARS-UCD1.2; Ensembl release 110) and BVDV genome (NCBI: ViralProj15305) using STAR (v2.7.10a) (28) with the following parameters: --alignIntronMax 1 \--chimOutType Junctions SeparateSAMold WithinBAM HardClip \--chimScoreJunctionNonGTAG 0 \--alignSJstitchMismatchNmax -1 -1 -1 -1 \-chimSegmentMin 12 \--chimJunctionOverhangMin 12 \--outSAMtype BAM SortedByCoordinate. Transcriptome clean reads were aligned to a combined reference comprising the bovine genome (ARS-UCD1.2, Ensembl release 110) and the BVDV genome (NCBI: ViralProj15305) using STAR (v2.7.10a) with the following parameters: --chimOutType Junctions SeparateSAMold WithinBAM HardClip \-chimScoreJunctionNonGTAG 0 \--alignSJstitchMismatchNmax -1 -1 -1 -1 \-chimSegmentMin 12. SAMtools (v1.21) and Picard (v3.3.0) were used to extract viral reads and reads chimeric between the virus and cattle, respectively.

### 2.7 RNA-sequencing and data processing

RNA integrity was assessed with the 2100 Bioanalyzer instrument (Agilent Technologies, Santa Clara, CA, USA) (**Supplementary Table 6**). Sequencing libraries were generated using Optimal Dual-mode mRNA Library Prep Kit (BGI-Shenzhen, China) following manufacturer’s recommendations and index codes were added to attribute sequences to each sample. Finally, the libraries were sequenced on DNBSEQ-T7 platform (BGI-Shenzhen, China) and 150 bp paired-end reads were generated.

We used SOAPnuke v2.1.9 (21) to filter out adapter sequences, low-quality bases/reads, and remove the first 14 bases with large base fluctuations in the reads. FastQC version 0.11.8 (http://www.bioinformatics.babraham.ac.uk/projects/fastqc/) was further used to evaluate the quality of the remaining reads. After the QC procedure is completed, the clean reads from each sample were aligned to the bovine reference genome (ARS-UCD1.2) and its corresponding gene annotations (https://jul2023.archive.ensembl.org/Bos_taurus/Info/Index) using STAR (version 2.7.10a) (28) in the two-pass mode. FeatureCounts (Subread version 2.0.1) (29) was used to quantify gene expression levels.

### 2.8 Differential expression analysis of genes

We performed differential expression analysis using gene count data as input data with the DESeq2 package in R (30). Differentially expressed genes (DEGs) were identified based on the criteria of adjusted *p* value < 0.05 and |log2 fold change| ≥ 1.

### 2.9 Differential expression analysis of transposable elements

We used the RepeatMasker software (version 4.1.7-p1) and the search engine Crossmatch (version 1.090518) to identify repetitive sequences by aligning the bovine reference genome (ARS-UCD1.2) against a library of known repeats (Dfam with RBRM Version: 3.8) (31). As recommended by TEtranscripts for preparing its input files, we aligned the clean sequencing reads to the reference genome (ARS-UCD1.2; Ensembl release 110) using STAR (v2.7.10a) (25) in two-pass mode with the parameters --winAnchorMultimapNmax 100 \--outFilterMultimapNmax 100. Subsequently, TEtranscripts was employed to quantify TE expression levels, leveraging gene annotation data and repetitive sequence annotation data produced by RepeatMasker (32). The Satellite/tRNA/snRNA/rRNA classes were excluded based on their nature as tandem repeats, while RC/Unknown/PLE elements were grouped as “other” due to their low abundance. Finally, differential expression analysis was performed using TE-only count data with the DESeq2 package (27), and identified differentially expressed transposable elements with adjusted *p* value < 0.05 and |log_2_ fold change| ≥ 1.

### 2.10 Functional enrichment analysis

We used the R clusterprofiler package (33) (https://www.bioconductor.org/packages/release/bioc/html/clusterProfiler.html) for GO and GSEA analyses. Briefly, we use the enrichGO function for GO enrichment analyses. For GSEA analysis, we sorted genes according to log_2_(foldchange) and conducted enrichment analysis using the gseGO functions.

### 2.11 Gene set variation analysis

The normalized expression (Transcripts Per Million, TPM) was calculated from gene counts using R (version 4.3.2), and ENSEMBL gene IDs were converted to SYMBOL IDs using the bitr function from the R package clusterProfiler. We downloaded the pathway data for *Bos taurus* from the KEGG database using the R package KEGGREST (https://www.bioconductor.org/packages/release/bioc/html/KEGGREST.html) and extracted immune system-related pathway information for GSVA analysis. We used the TPM data of the genes as input data, applied the R package GSVA to calculate pathway scores for each sample, and performed Wilcoxon tests to assess differences between groups.

### 2.12 Protein-protein interaction analysis

We utilized the STRING database (https://cn.string-db.org/) to explore protein-protein interactions among the genes. Specifically, we uploaded the gene IDs of significantly differentially expressed genes and potential methylation-regulated genes using the “Multiple proteins” mode and performed the analysis with the default parameters to identify potential interactions.

### 2.13 Deconvolution analysis of bulk RNA-seq data

The deconvolution analysis was performed with CIBERSORTx (34). Among them, cow peripheral blood single cell transcriptome data comes from the study of Zhang et al. (35) (GSM5368460). Following the CIBERSORTx tutorial, we input the single cell count data to obtain the signature matrix, and then we input our bulk RNA-seq count data to perform deconvolution and obtain the cell proportion data for each sample. For subsets from the same cell type, we aggregated them to obtain the proportion represented by the major cell type and performed Wilcoxon tests to assess differences between groups.

### 2.14 Reduced representation bisulfite sequencing and data processing

After confirming DNA quality, a total amount of 5.2 microgram genomic DNA spiked with 26 ng lambda was digested using methylation-insensitive restriction enzyme Mspl. DNA were fragmented by sonication to 200–300 bp with Covaris S220 (Covaris, USA), followed by end repair and adenylation. Cytosine-methylated barcodes were ligated to sonicated DNA following the manufacturer’s manual. Then these DNA fragments were treated twice with bisulfite using EZ DNA Methylation-GoldTM Kit (Zymo Research, USA, Catalog #: D5005), before the resulting single-strand DNA fragments were PCR amplificated using KAPA HiFi HotStart Uracil + ReadyMix (2X). Subsequently, library quality was assessed on the Agilent 5400 system (Agilent, USA) and quantified by QPCR (1.5 nM). Finally, the libraries were sequenced on Illumina platforms and 150 bp paired-end reads were generated (Novogene Bioinformatics Technology, Beijing, China).

The quality and adapter trimming of Illumina raw sequences were performed using Trim Galore in RRBS mode (https://github.com/FelixKrueger/TrimGalore?tab=readme-ov-file). Subsequently, FastQC version 0.11.8 (http://www.bioinformatics.babraham.ac.uk/projects/fastqc/) was used to evaluate the quality of the remaining reads. The clean data were aligned to the bovine reference genome (ARS-UCD1.2) using Bismark (36) with the option -N 1 (maximum number of mismatches allowed), and the resulting files were used in MethPipe (v5.0.1) for subsequent analysis.

### 2.15 Methylation analysis

We focused on DNA methylation at CpG sites and retaining only those sites that 1) were present across all samples and 2) had a coverage of ≥10 reads per sample. After filtration, 1,417,600 CpG cytosines were retained for subsequent analysis.

We identified hypomethylated and hypermethylated regions using the hmr or hmr-rep program in MethPipe with default parameters. Bedtools intersect (37) with -F 0.5 was used to identify transposable elements (TEs) overlapping with hypomethylated regions, with overlaps defined as covering more than 50% of either the hypomethylated region or the TE.

The differentially methylated sites of the “PI calf group versus normal calf group” and “Trojan dam group versus normal dam group” were identified using the radmeth program in MethPipe with the default parameters. Significance combining and multiple testing correction were performed using the radadjust program. The output file contains 11 columns: the first four columns represent the genomic coordinates of each CpG site; the fifth column contains the raw *p* value; the sixth column shows the modified *p* value, which is adjusted based on the site’s original *p* value and the *p* values of its neighboring sites; and the seventh column provides the FDR-corrected *p* value. The final four columns correspond to the total read counts and methylated read counts for the case and control groups, respectively. Differentially methylated regions (DMRs) were identified using MethPipe’s radmerge program with the option -p 0.05, utilizing the output from radadjust as input.

To identify methylation regions unique to PI calves, we first conducted differential methylation analyses by comparing PI calves with normal calves, normal dams, and Trojan dams, respectively. CpG sites that were significantly different across all three pairwise comparisons and exhibited the same direction of change were defined as PI-calf–specific methylation sites. The radmerge program from MethPipe was used to cluster PI-calf–specific methylation sites into differentially methylated regions (DMRs).

The identified differentially methylated regions (DMRs) were subjected to further filtering prior to downstream analysis based on the following criteria: 1) containing ≥ 3 CpG sites and 2) exhibiting an absolute methylation difference ≥ 0.025.

### 2.16 Identification of genes associated with differentially methylated regions and transcription factor motif analysis

Genes whose promoter regions (defined as 2 kb upstream and downstream of the transcription start sites) and genebody regions overlapped with DMRs were identified using bedtools intersect with default parameters, without considering strand specificity. Based on the well-established mechanism by which promoter methylation modulates transcription factor binding and affects gene expression, high methylation levels can reduce transcription factor accessibility and decrease gene expression (38–41). Genes whose promoters contained differentially methylated regions (DMRs) and showed significant differential expression (adjusted *p* value < 0.05), with opposite directions of change between promoter methylation and gene expression, were considered potentially regulated by methylation. Transcription factor (TF) motif scanning was performed on differentially methylated regions (DMRs) using HOMER’s annotatePeaks.pl program (35) with its vertebrate TF database with the following parameters: annotatePeaks.pl dmr.bed reference.fa \-gtf file.gtf \-norevopp \-m known.motifs \-nmotifs \-mdist.

## 3 Results

### 3.1 Overview of samples

**Figure 1:**
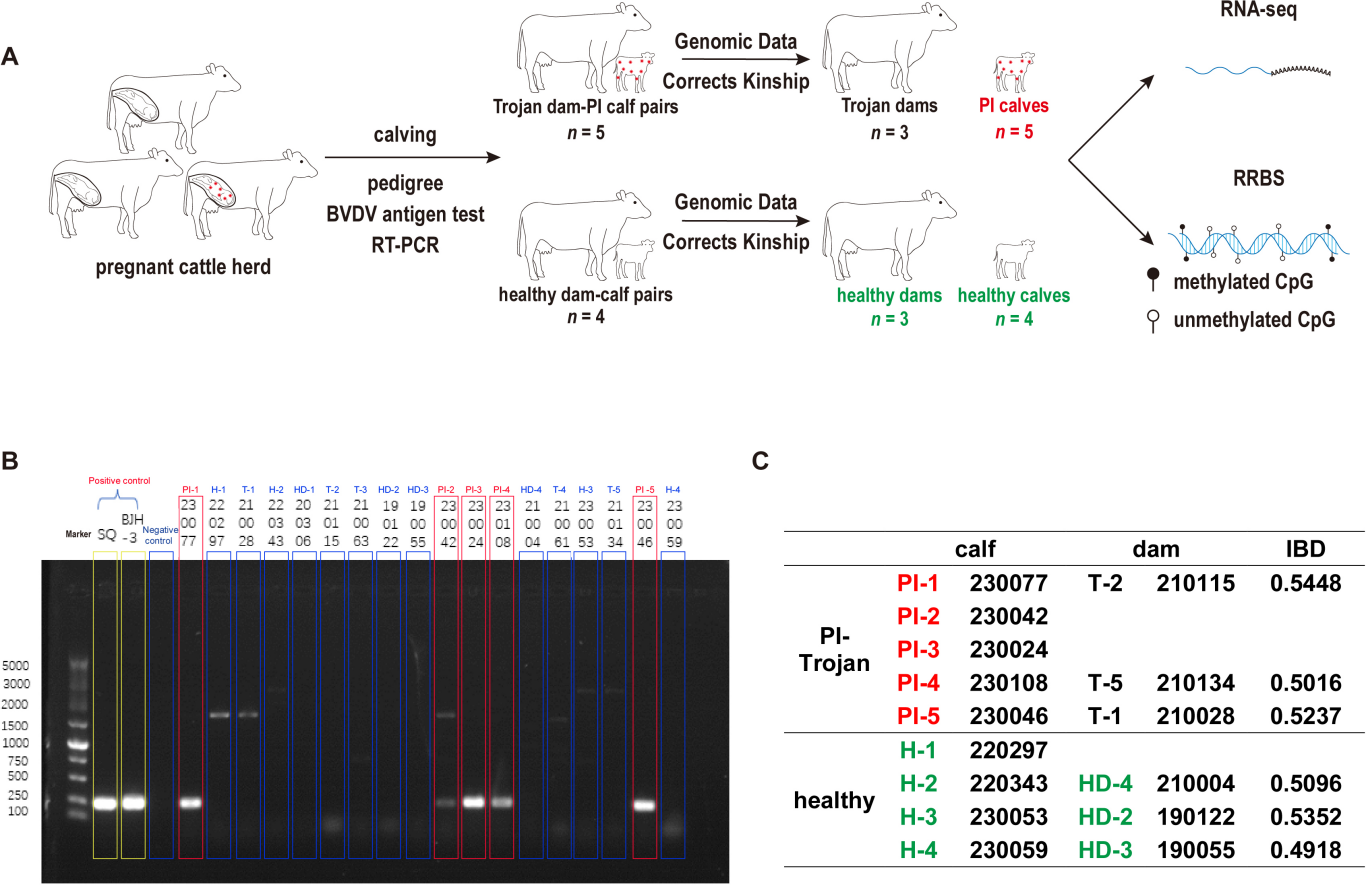
Experimental Design. **(A)** Workflow of experimental. **(B)** BVDV detection by RT-PCR. Yellow and Red boxes denote samples testing positive, while blue boxes denote samples testing negative. PI, persistently infected calf H, healthy calf; T, trojan dam; HD, healthy dam; The number label (e.g., 230077): farm cattle ID. **(C)** Genomic data correct kinship.

In this study, we collected samples from four pairs of healthy dam-calf and five pairs of Trojan dam-PI calf, and performed genome, transcriptome, and methylome sequencing (**Figure 1A**). To further confirm BVDV infection, we performed reverse transcription polymerase chain reaction (RT-PCR) on serum samples from all individuals. As expected, only the PI calves tested positive, whereas normal calves, normal dams, and Trojan dams tested negative (**Figure 1B**).

To avoid errors in pedigree information, we used genomic data to calculate IBD and perform pedigree inference for the dam-calf pairs. The results showed that only 3 healthy dam-calf pairs and 3 Trojan dam-PI calf pairs had IBD and pedigree inference results consistent with the mother-daughter relationship (**Figure 1C** and **Supplementary Table 1**). Therefore, all PI calves, normal calves, and their genetically confirmed dams will be included in subsequent analyses (**Figure 1A**).

### 3.2 Transcriptome analysis

**Figure 2:**
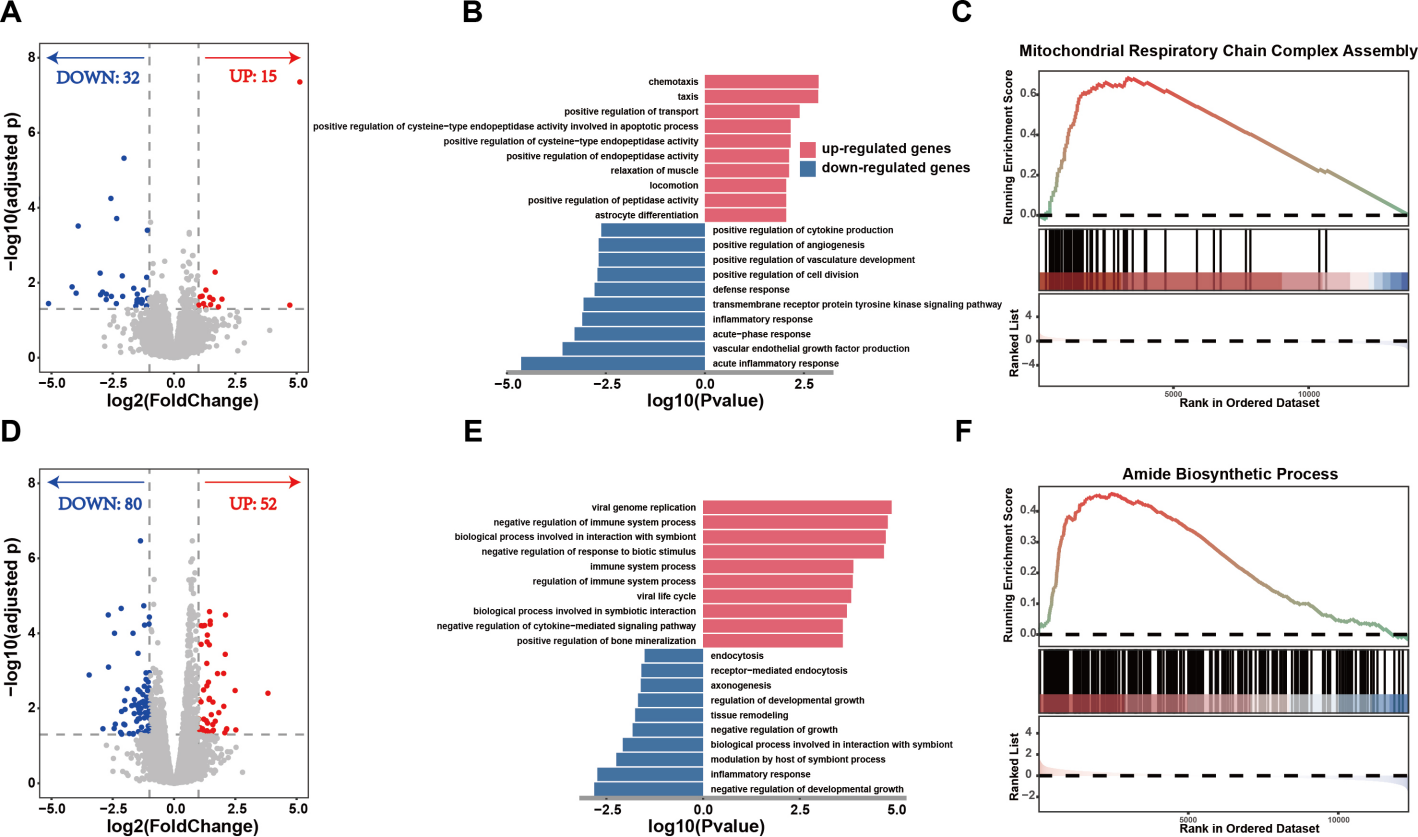
Transcriptome differential expression analyses. **(A)** Volcano plot of differential expression analysis of calf groups. **(B)** GO enrichment pathways of significantly differentially expressed genes in calf groups (*p* < 0.05). **(C)** Gene set enrichment analysis revealed that the Mitochondrial Respiratory Chain Complex Assembly pathway was significantly upregulated in in persistent infection calves (adjusted *p* < 0.05). **(D)** Volcano plot of differential expression analysis of dam groups. **(E)** GO enrichment pathways of significantly differentially expressed genes in dam groups (*p* < 0.05). **(F)** Gene set enrichment analysis revealed that the Amide Biosynthetic Process pathway was significantly upregulated in in persistent infection calves (adjusted *p* < 0.05).

Both PI and normal calves appear clinically normal, but PI calves are more susceptible to diseases and grow slower, suggesting potentially distinct intrinsic molecular regulatory networks. To investigate differences in gene regulatory networks, we performed transcriptome analysis and found 15 significantly upregulated genes (log_2_ (fold change) ≥ 1 and adjusted *p* < 0.05) and 32 significantly downregulated genes (log_2_ (fold change) ≤ -1 and adjusted *p* < 0.05) in PI calves compared to normal calves (**Figure 2A**). Gene Ontology (GO) enrichment analysis revealed that the significantly upregulated genes were mainly enriched in pathways related to peptidase regulation, including the positive regulation of cysteine-type endopeptidase activity involved in apoptosis, positive regulation of cysteine-type endopeptidase activity, and others (**Figure 2B**). The significantly downregulated genes were primarily enriched in inflammation-related biological processes, including acute inflammatory response, positive regulation of cytokine production (**Figure 2B**). Biological pathways involve the coordinated regulation of multiple genes rather than being driven by a few. Therefore, we performed Gene Set Enrichment Analysis (GSEA) to investigate overall biological pathway differences between PI calves and normal calves. Compared to normal calves, PI calves showed significant upregulation in energy metabolism-related pathways, including mitochondrial respiratory chain complex assembly and peptide metabolic processes, while exhibiting significant downregulation in inflammation-related pathways such as acute inflammatory response and interleukin-2 production (**Figure 2C**, **Supplementary Figure 1A** and **Supplementary Table 3**).

Trojan cattle carried PI fetuses during pregnancy but remained clinically normal, suggesting that their gene regulatory networks may have undergone significant changes to counteract viral threats compared to those of normal mothers. We found that 80 genes were significantly downregulated and 52 genes were significantly upregulated in Trojan cattle compared to normal cattle (**Figure 2D**). The upregulated gene sets were predominantly enriched in biological processes related to viral genome replication, immune system activity, and other pathways associated with viruses and immunity (**Figure 2E**). The downregulated gene sets were mainly enriched in biological processes such as inflammatory response, modulation by host of symbiont process, and others (**Figure 2E**). Furthermore, GSEA analysis revealed that Trojan cattle showed significant upregulation in biological processes such as amide and peptide biosynthesis and metabolism, while showing significant downregulation in processes such as chemokine production and regulation (**Figure 2F**, **Supplementary Figure 1B**, **Supplementary Table 4**).

### 3.3 Impaired immune system in PI cattle

**Figure 3:**
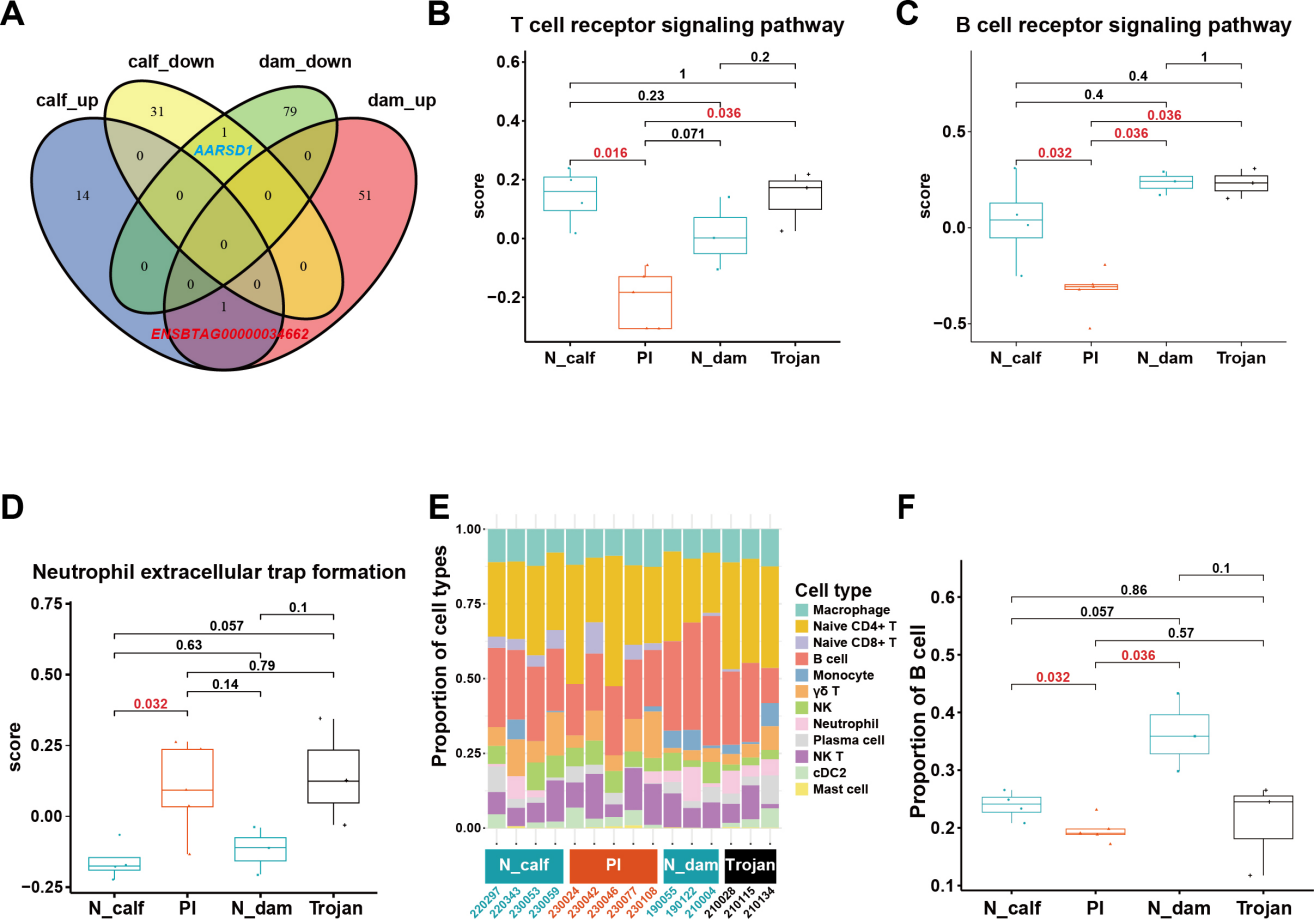
GSVA and single-cell deconvolution analysis. **(A)** Venn diagram of significantly differentially expressed genes between the calf groups and the dam groups. calf_up: Genes significantly upregulated in PI calves compared with normal calves; calf_down: Genes significantly downregulated in PI calves compared with normal calves; dam_up: Genes significantly upregulated in trojan dams compared with normal dams; dam_down: Genes significantly downregulated in trojan dams compared with normal dams. **(B-D)** GSVA scores of immune-related pathways in each group. **(E)** Proportions of 12 cell types obtained by CIBERSORTx deconvolution. **(F)** Differences in B cell proportions among different groups. N_calf, normal calf group; PI, persistently infected **(PI)** calf group; N_dam, Dam group of normal calves; Trojan, Dam group of PI calves.

To identify conserved molecular changes between PI calves and Trojan dams, we analyzed the shared differentially expressed genes and enriched biological pathways in the comparisons of “PI calves vs. normal calves” and “Trojan dams vs. normal dams”. *AARSD1* was significantly downregulated, while *ENSBTAG00000034662* was significantly upregulated in both PI calves and Trojan dams (**Figure 3A**). According to GSEA results, biological pathways such as amide/peptide biosynthetic processes (GO:0043604, GO:0043043) and metabolic processes (GO:0006518, GO:0043603) were significantly upregulated (**Supplementary Tables 3**, **4**).

Furthermore, we conducted Gene Set Variation Analysis (GSVA) to investigate the differences in immune system-related biological pathways among the four groups: PI calves, normal calves, Trojan dams, and normal dams. Compared with other groups, PI calves exhibited significant downregulation (*p* < 0.05) in key immune pathways, including T cell receptor signaling pathway, B cell receptor signaling pathway, and other critical pathways (**Figures 3B, C**, **Supplementary Figure 2**). Notably, we observed upregulation of the neutrophil extracellular trap formation pathway in Trojan dam-PI calf pairs relative to healthy dam-calf pairs, though this trend did not reach statistical significance (*p* < 0.05) when comparing Trojan dams to healthy dams alone (**Figure 3D**). Meanwhile, single-cell deconvolution analysis indicated that PI calves exhibited a significantly reduced B cell proportion relative to normal calves (*p* = 0.032, **Figures 3E, F**). Together, these results suggest that viral infection may compromise the immune function of PI calves, resulting in an overall state of immunosuppression.

### 3.4 DNA methylation influences the status of PI calves

**Figure 4:**
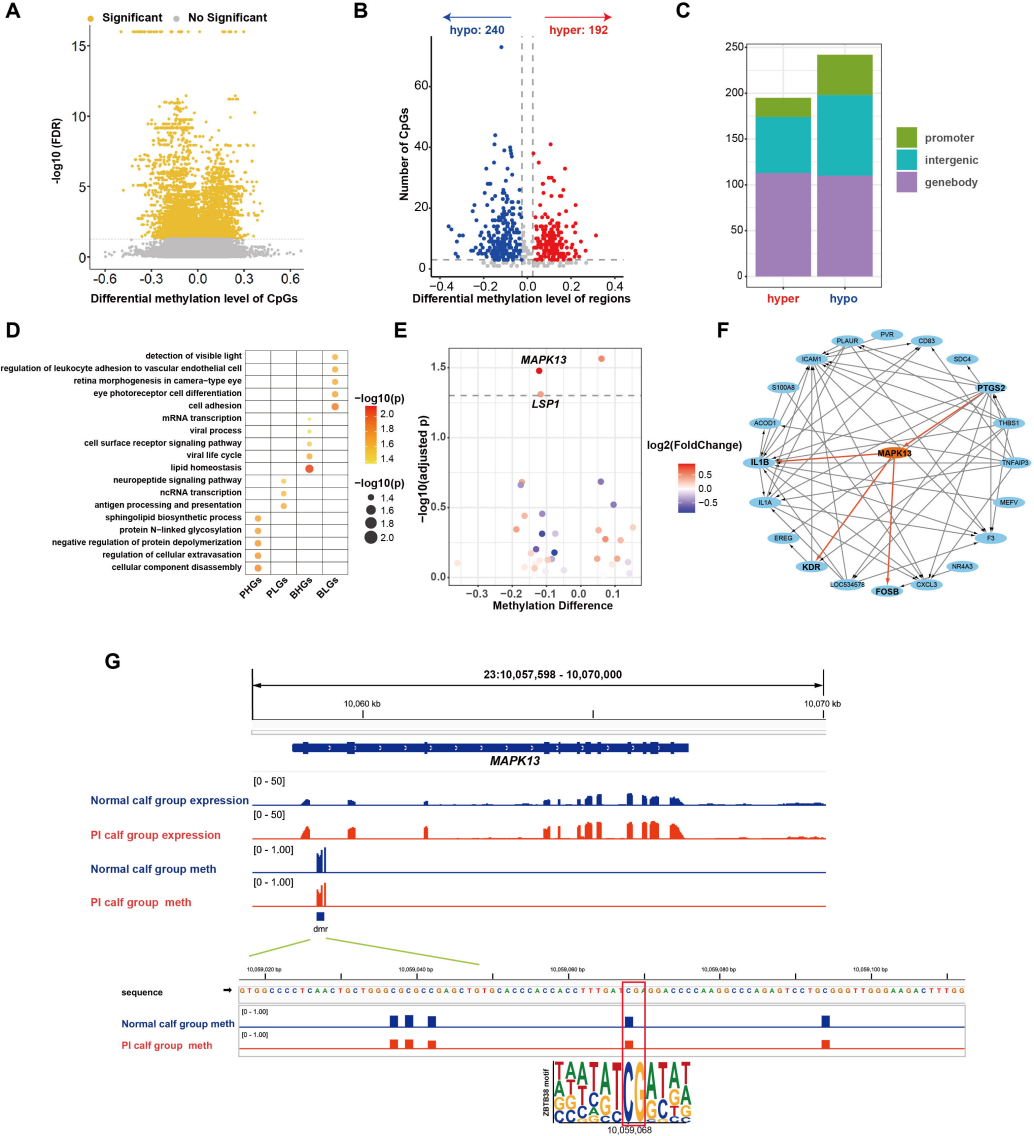
Methylation analyses of calf groups. **(A)** Volcano plot of differentially methylated sites. **(B)** Volcano plot of DMRs. **(C)** Distribution of DMRs on the genome. **(D)** Significantly enriched GO terms across gene categories (*p* < 0.05). **(E)** Associations between gene expression differences and corresponding DMR methylation differences of genes containing DMRs in their promoter regions. **(F)** PPI network between genes with expected methylation-expression relationships and significantly differentially expressed genes in the calf groups. **(G)** The DMR in the *MQPK13* promoter region and its putative transcription factor. Normal calf group expression: IGV tracks showing average RNA-seq signals (TPM) at the *MAPK13* locus in the normal calf group; PI calf group expression: IGV tracks showing average RNA-seq signals (TPM) at *MAPK13* locus in PI calf group; Normal calf group meth: IGV tracks showing average methylation levels at *MAPK13* locus in normal calf group; PI calf group meth: IGV tracks showing average methylation levels at *MAPK13* locus in PI calf group. ZBTB38 motif: Base sequences matching ZBTB38 motifs within DMRs that contain significantly differentially methylated CpG sites.

Regardless of genetic background, calves born to dams infected with BVDV during a specific gestational window develop persistent infection (PI), characterized by lifelong viral shedding and immune dysfunction (8, 10, 12). These state alterations may be mediated by virus-induced epigenetic changes (20, 42, 43); therefore, we further investigated methylome differences between PI calves and normal calves. We identified 5,332 significantly differentially methylated CpG sites (FDR < 0.05) in PI calves compared with normal calves (**Figure 4A**). We further used the MethPipe pipeline, which yielded a total of 432 differentially methylated regions (DMRs), including 240 regions with lower methylation and 192 regions with higher methylation in PI calves compared with normal calves (**Figure 4B**). The majority of DMRs were located in genebody regions, followed by intergenic regions, while only a few were mapped to promoter regions (**Figure 4C**).

As methylation in promoter or genebody regions may influence gene expression, we then investigated the potential functional of genes associated with these DMRs. Genes were categorized into four groups based on DMR location (promoter or genebody) and methylation state (higher or lower methylation in PI calves), followed by Gene Ontology (GO) enrichment analysis for each group. Promoter higher methylation genes (PHGs) showed enrichment in biosynthetic pathways, such as cellular component disassembly and negative regulation of protein depolymerization, whereas promoter lower methylation genes (PLGs) were mainly enriched in antigen processing and presentation (**Figure 4D**). Genebody higher methylation genes (BHGs) were predominantly enriched in homeostasis- and virus-associated pathways, such as lipid homeostasis and viral life cycle, whereas genebody lower methylation genes (BLGs) were mainly enriched in pathways such as cell adhesion (**Figure 4D**). Given the well-established mechanism by which promoter methylation regulates gene expression through modulation of transcription factor binding, we focused on genes harboring DMRs in their promoters for subsequent investigation. By integrating methylation and transcriptome data, we identified two genes (*MAPK13*, *LSP1*) whose promoter methylation affected gene expression as expected (**Figure 4E**). We further performed protein-protein interaction (PPI) analysis between these genes and significantly differentially expressed genes to investigate the potential cascade reactions induced by methylation-affected genes. We found that *MAPK13* may potentially influence the expression of *IL1B*, *KDR*, *FOSB*, and *PTGS2*, thereby impacting the physiological and biochemical status of cattle (**Figure 4F**). We further conducted a transcription factor motif scan on the DMRs within the *MAPK13* and *LSP1* promoter. This analysis revealed that the differentially methylated sites overlap with the binding motif for ZBTB38, suggesting that methylation may modulate its binding affinity and thereby influence *MAPK13* expression (**Figure 4G**).

We further investigated whether DNA methylation might drive aberrant transposon expression and thereby facilitate potential BVDV integration into the host genome. A comparison of the number of transposons located within hypomethylated regions (HMRs) revealed no significant differences (*p* > 0.05) between PI calves and normal calves (**Supplementary Figures 3A, B**). Similarly, transposon expression levels did not differ significantly between the two groups (**Supplementary Figure 3C**). In addition, we aligned DNA isolated from the blood of PI cattle to the BVDV reference genome and analyzed RNA-seq data to detect potential chimeric transcripts. Both analyses revealed no evidence of BVDV sequences in the bovine genome.

### 3.5 Persistent DNA methylation features imprinted by BVDV infection in dams

**Figure 5:**
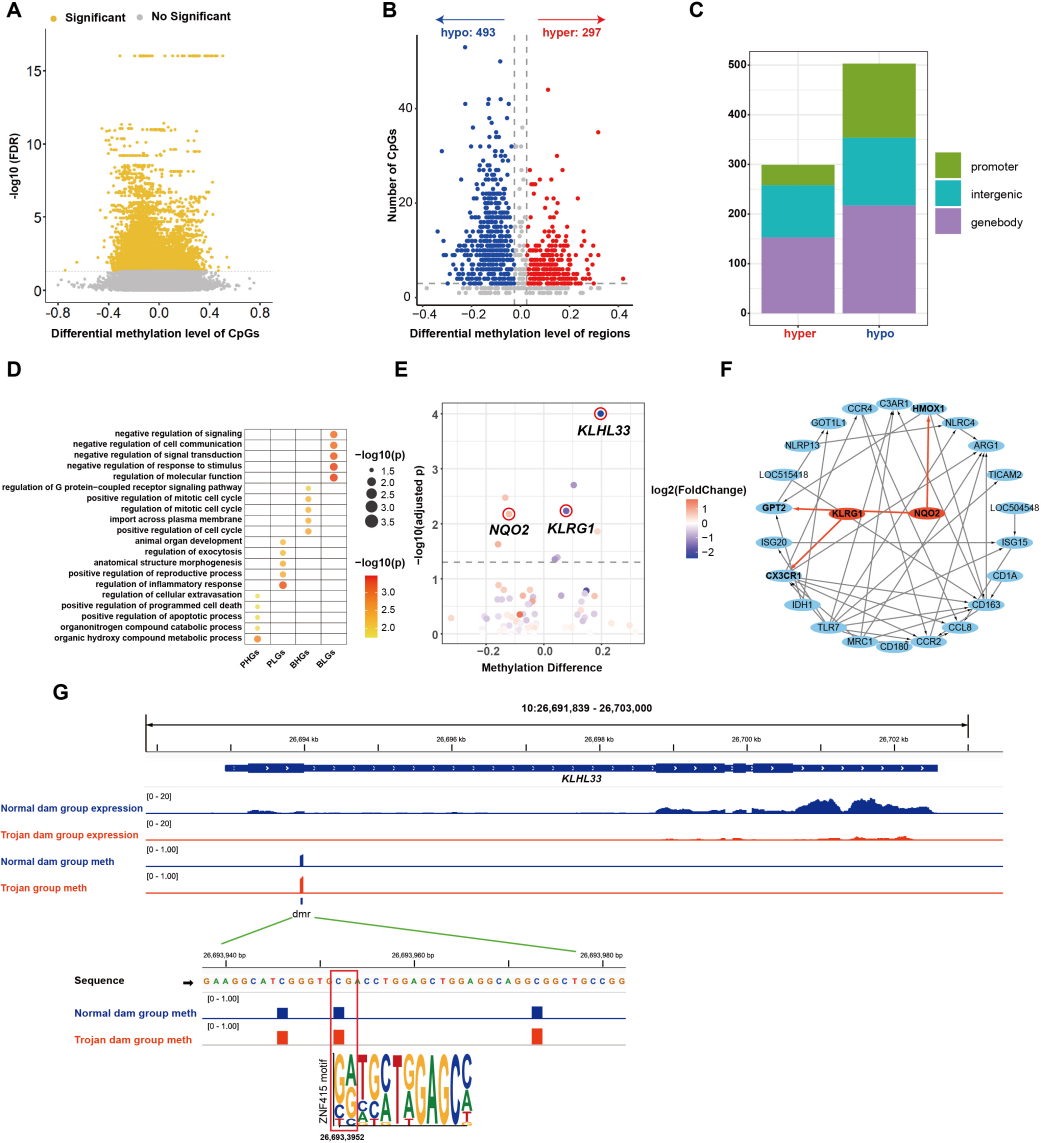
Methylation analyses of dam groups. **(A)** Volcano plot of differentially methylated sites. **(B)** Volcano plot of DMRs. **(C)** Distribution of DMRs on the genome. **(D)** Significantly enriched GO terms across gene categories (*p* < 0.05). **(E)** Associations between gene expression differences and corresponding DMR methylation differences of genes containing DMRs in their promoter regions. **(F)** PPI network between genes with expected methylation-expression relationships and significantly differentially expressed genes in the dam groups. **(G)** The DMR in the *KLHL33* promoter region and its putative transcription factor. Normal dam group expression: IGV tracks showing average RNA-seq signals (TPM) at the *KLHL33* locus in the normal dam group; Trojan dam group expression: IGV tracks showing average RNA-seq signals (TPM) at *KLHL33* locus in Trojan dam group; Normal dam group meth: IGV tracks showing average methylation levels at *KLHL33* locus in Normal dam group; Trojan group meth: IGV tracks showing average methylation levels at *KLHL33* locus in Trojan group. ZNF415 motif: Base sequences matching ZNF415 motifs within DMRs that contain significantly differentially methylated CpG sites.

We identified a total of 9,812 significantly differentially methylated CpG sites (FDR < 0.05) in the dam groups (**Figure 5A**). These significant CpG sites were combined into 790 DMRs (**Figure 5B**). Among these, 493 were lower methylation and 297 were higher methylation in Trojan dams compared with the normal dams (**Figure 5B**). Similar to the calf groups, DMRs were predominantly located in genebody regions, followed by intergenic regions, with the lowest proportion found in promoters (**Figure 5C**).

We further performed GO enrichment analysis on genes overlapping with DMRs and observed that distinct gene categories were enriched in different biological processes. Promoter higher methylation genes (PHGs) are primarily enriched in biosynthesis- and cell death-related pathways, such as organic hydroxy compound metabolism and positive regulation of apoptosis, whereas promoter lower methylation genes (PLGs) are mainly enriched in regulating the inflammatory response pathway (**Figure 5D**). Genebody higher methylation genes (BHGs) are mainly enriched in cell cycle-related pathways, such as positive regulation of the cell cycle, whereas genebody lower methylation genes (BLGs) are enriched in signal transduction-related pathways, including negative regulation of response to stimulus and negative regulation of signal transduction (**Figure 5D**). To identify genes potentially regulated by methylation, we integrated methylation and transcriptome data and identified eight genes whose promoter methylation affected their expression as anticipated (**Figure 5E**). Four of the eight genes were significantly differentially expressed (|log2 FoldChange| ≥ 1 and adjusted *p* < 0.05), with *KLHL33* showing the most substantial changes in both methylation and transcriptional levels (**Figure 5E**, **Supplementary Table 2**). PPI network analysis suggested that *NQO2* may induce significant differential expression of *GPT2* and *HMOX1* through cascade reactions, whereas *KLRG1* may affect *CX3CR1* (**Figure 5F**). Furthermore, we performed a transcription factor motif scan on the DMRs of these eight genes and identified potential binding transcription factors, such as ZNF415 binding sites on *KLHL33* that harbor differentially methylated CpG site (**Figure 5G**, **Supplementary Table 5**).

### 3.6 Identification of DNA methylation markers of PI calves

**Figure 6:**
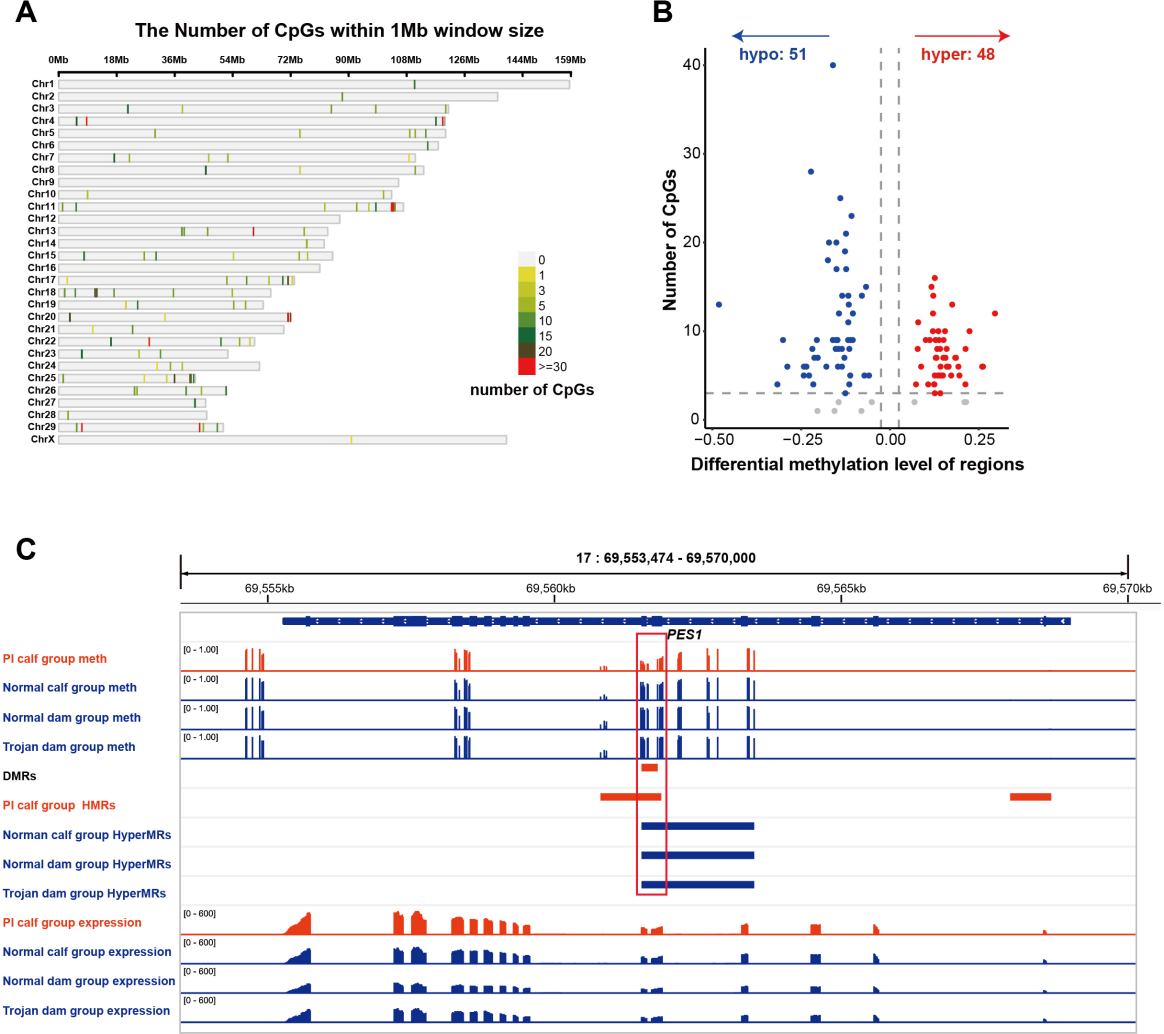
PI calf-specific DNA methylation markers. **(A)** Distribution of significantly differentially methylated sites in the genome. **(B)** Volcano plot of DMRs. **(C)** Methylation patterns and gene expression levels of the *PES1* gene across different groups. PI calf/Normal calf/Normal dam/Trojan dam group meth: IGV tracks showing average methylation levels at the *PSE1* locus across groups. PI calf group HMRs: IGV tracks showing hypomethylated regions at the *PSE1* locus in the PI calf group; Normal calf/Normal dam/Trojan dam group HyperMRs: IGV tracks showing hypermethylated regions at the *PSE1* locus across groups; DMR: IGV tracks of PI-specific differentially methylated regions (DMRs) at the *PSE1* locus; PI calf/Normal calf/Normal dam/Trojan dam group expression: IGV tracks showing average RNA-seq signals (TPM) at the *PES1* locus across groups; red boxed: PI-specific methylation region at the *PSE1* locus.

We compared the methylation data of the PI calf group against each of the other three groups separately and then integrated the results to identify differentially methylated CpG sites that were consistently higher or lower methylation across all comparisons. A total of 993 CpG sites were identified, with the vast majority located on autosomes and only a single site found on the X chromosome (**Figure 6A**). Among them, 400 sites were significantly higher methylation in PI calves, and 593 sites were significantly lower methylation. The significantly differentially methylated sites were subsequently grouped into 99 DMRs, of which 48 were higher methylation and 51 were lower methylation in the PI group relative to the other groups (**Figure 6B**). Six DMRs exhibited methylation differences greater than 0.25, with the *PES1* gene region showing the largest difference, approaching 0.5 (**Figure 6B**). Notably, this DMR is located in the HMR of PI calves but in hypermethylated region (HyperMR) of the other groups, highlighting its potential as a candidate diagnostic biomarker for PI cattle detection (**Figure 6C**). Meanwhile, we observed a trend toward increased expression of *PES1* in the PI calf group compared with the other groups, which may potentially be regulated by this DMR (**Figure 6C**).

## 4 Discussion

BVDV poses a significant threat to the global cattle industry, with prevention serving as the primary strategy for its control (5, 44, 45). The PI cattle are the primary source of infection within herds, and elucidating their mechanisms of immunosuppression as well as identifying novel biomarkers is crucial for improving BVDV prevention and control strategies. Although PI cattle appear clinically healthy, our transcriptome analysis revealed reduced immune competence in these animals. By integrating transcriptomic and methylation data, we identified that differential methylation of the *MAPK13* promoter may underlie its aberrant expression in PI cattle. In Trojan dams, the abnormal expression of genes including *KLHL33* and *NQO2* was found to be potentially regulated by DNA methylation. Furthermore, comparative analysis revealed 99 DMRs between PI calves and the other groups, including a region in the *PES1* gene that was specifically hypomethylated in PI calves, highlighting its potential as a candidate diagnostic biomarker.

Fetal infection with BVDV prior to immune system maturation results in the inability to mount a competent immune response, leading to immunotolerance and lifelong viral persistence (8, 46–48). The majority of PI cattle exhibit stunted growth and increased susceptibility to secondary infections (10, 47). Our transcriptome analysis showed that PI calves had significantly reduced activity in immune-related pathways, such as B cell receptor and T cell receptor signaling pathways. B cells and T cells are key components of the adaptive immune response. Antigen presenting cells (APCs) present exogenous antigens on MHC class II molecules, which are recognized by CD4^+^ T cell receptor (TCR), triggering their activation and differentiation into effector T cells that orchestrate adaptive cellular immune responses (49–51). Upon antigen binding by B cell receptor (BCR) and with T helper (Th) cell assistance, B cells become fully activated and differentiate into antibody-secreting plasma cells that initiate the humoral immune response (51–53). Therefore, reduced TCR and BCR signaling activities suggest an impaired adaptive immune response in PI cattle, limiting their ability to mount effective antiviral immunity and increasing susceptibility to secondary infections. Furthermore, single-cell deconvolution of bulk RNA-seq data from bovine blood revealed that PI calves had a significantly lower proportion of B cells. Taken together, these findings reveal molecular-level immune impairment in PI calves despite their clinically normal phenotype.

The regulation of gene expression is shaped by the combined effects of the genome and the epigenome. BVDV, as a positive-stranded RNA virus that lacks the intrinsic ability to integrate into the host genome, is therefore more likely to modulate gene expression by influencing specific epigenetic modifications (1, 20, 54). Therefore, we mainly focused on whether alterations in the epigenome, specifically DNA methylation, regulate gene expression and consequently affect the physiological and biochemical status of PI cattle. We identified a total of 5,332 significantly differentially methylated CpG sites between PI calves and normal calves, which were consolidated into 432 DMRs. Integrative analysis of transcriptome and methylome data revealed that the expression of *MAPK13* and *LSP1* is potentially regulated by DNA methylation, with lower promoter methylation level corresponding to higher gene expression level in PI cattle. As a component of the p38 MAPK pathway, *MAPK13* plays a role in stress responses and innate immunity, but excessive expression can enhance cytokine production and maintain inflammatory states (55–58) (**Table 1**). The *LSP1* encoded protein serves as a substrate of mitogen-activated protein kinase-activated protein kinase 2 (MK2) in the p38 MAPK–MK2–LSP1 signaling pathway, and its phosphorylation regulates leukocyte proliferation and differentiation, as well as neutrophil migration and chemotaxis (59, 60). Concurrently, protein-protein interaction analysis of *MAPK13* with significantly differentially expressed genes indicates that *MAPK13* may influence downstream genes through cascade reactions, ultimately affecting immune and inflammatory processes. For example, *IL1B*, a potential downstream target of *MAPK13*, belongs to the interleukin-1 family, and its elevated expression may contribute to autoinflammatory or chronic diseases (61). In general, *MAPK13* potentially orchestrates changes in PI calves by both directly regulating transcriptional programs and promoting LSP1 phosphorylation through its cooperative interaction with elevated LSP1, thereby disrupting physiological homeostasis. DNA methylation in promoter regions generally regulates gene expression by affecting transcription factor binding (39). Through transcription factor motif scanning, we identified ZBTB38 as a potential binding transcription factor within the differentially methylated regions of *MAPK13*. Additionally, although BVDV itself cannot integrate into the host genome, previous studies have demonstrated that positive-stranded RNA virus (severe acute respiratory syndrome coronavirus 2) can become integrated into host genomes via a LINE1-mediated retrotransposition mechanism (27). DNA methylation is a critical epigenetic modification that silences transposable elements; reduction in methylation levels can result in their aberrant expression (62). Although our analysis revealed no viral integration altering the host genome, we acknowledge that a sequencing depth of 10x may be insufficient to detect integration events. Future studies combining high-depth resequencing and long-read sequencing technologies are needed to determine whether BVDV integrates into the host genome.

**Table 1:** Functions of key genes identified in this study.

Gene	Full Name	Function	Ref
*MAPK13*	mitogen-activated protein kinase 13	A member of the p38 MAPK family involved in stress responses and innate immunity	(55–58)
*LSP1*	lymphocyte specific protein 1	Regulates leukocyte proliferation/differentiation and neutrophil migration/chemotaxis	(59, 60)
*IL1B*	interleukin 1 beta	A key pro-inflammatory cytokine that mediates immune and inflammatory signaling	(61, 79, 80)
*AARSD1*	alanyl-tRNA synthetase domain containing 1	Ensuring the accurate utilization and translation of genetic information	(64)
*KLHL33*	kelch like family member 33	Substrate adaptors of Cullin 3–RING ligases (CRL3), mediating ubiquitination and degradation of target proteins	(81, 82)
*NQO2*	N-ribosyldihydronicotinamide:quinone dehydrogenase 2	Catalyzes two-electron reductions of quinones, pseudoquinones	(83–85)
*PES1*	Pescadillo ribosomal biogenesis factor 1	Involved in cancer initiation and progression; potential biomarker and therapeutic target	(86–89)

Trojan dams are infected with BVDV during pregnancy, which elicits a normal immune response leading to antibody production, after which they recover (63). Notably, we found that AARSD1 was significantly downregulated in both PI and Trojan cattle, whereas ENSBTAG00000034662 was markedly upregulated. The *AARSD1* gene encodes the alanyl-tRNA editing protein Aarsd1, which plays a critical role in ensuring the accurate utilization and translation of genetic information (64). Simultaneously, both PI and Trojan cattle exhibited elevated activity in the neutrophil extracellular trap formation pathway. Neutrophil extracellular traps (NETs) are DNA-protein complexes that help resist pathogens but can also cause tissue damage and promote inflammation when produced excessively (65–67). These findings indicate that BVDV infection may have long-lasting effects on Trojan dams.

Elimination of PI animals is crucial for controlling BVDV infection in cattle (68, 69). While Trojan dams can be identified via antibody levels, this method lacks sufficient sensitivity and specificity for practical use, and direct viral antigen detection in amniotic or allantoic fluid via intrauterine puncture is costly and difficult to implement widely (10). Therefore, the most practical approach for testing PI cattle remains diagnosing calves after birth and typically followed by a second test two weeks later. DNA methylation not only regulates gene expression but also serves as a reliable biomarker for disease detection (70, 71). Here, we identified 9,812 significantly differentially methylated CpG sites between Trojan dams and normal dams, which were consolidated into 790 DMRs. A total of eight genes were identified whose expression is potentially regulated by DNA methylation. These changes in Trojan dams-specific epigenetic modifications may reflect immune memory induced by BVDV infection, facilitating responses to subsequent challenges (72). Infection with BVDV before day 125 of gestation leads to persistent infection in the fetus, may remain persistent between days 125 and 150 depending on individual differences in immune development, and becomes only transient after day 150 (7, 8, 11, 73). We can use Trojan dam-specific DNA methylation markers for preliminary identification after 150 days of gestation. Furthermore, we compared PI calves with the other groups and identified 993 significantly differentially methylated sites, which were aggregated into 99 DMRs. Among these, the DMR located in the *PES1* gene showed the greatest differential methylation, which was located in HMR in PI calves and in HperMR in the other groups, indicating its potential utility as candidate diagnostic biomarker for identifying PI cattle. Cell-free DNA (cfDNA), generated through cell death or active secretion and released into body fluids, carries abundant genetic information such as methylation and fragment profiles, enabling early non-invasive fetal diagnosis and disease monitoring (74–78). We can further extract cfDNA from candidate Trojan dams to assay for PI-specific DNA methylation markers, thereby enhancing the specificity and sensitivity of prenatal PI screening.

We acknowledge the limitations of our study, including the small sample size and the need for further validation of these methylation biomarkers in practical applications. Nevertheless, this work can be considered a proof-of-concept study, providing a foundational perspective for advancing prenatal screening of PI cattle. Future studies will aim to expand the sample size and employ whole genome methylation sequencing to comprehensively identify specific methylation markers in Trojan dams and PI calves, as well as to rigorously validate the reliability of DNA methylation markers as biomarkers for prenatal diagnosis of PI cattle. Collectively, our study systematically assessed the molecular-level changes in PI calves and Trojan dams relative to normal cattle, identifying *MAPK13* as a potential key gene underlying the subclinical condition of PI calves. Additionally, our study identified a DMR located in the *PES1* gene that is hypomethylated in PI calves but hypermethylated in other groups, highlighting its potential as a candidate diagnostic biomarker for PI detection.

## Data Availability

All sequencing raw data used in this study have been uploaded in the Genome Sequence Archive in National Genomics Data Center, China National Center for Bioinformation/Beijing Institute of Genomics, Chinese Academy of Sciences (GSA: CRA037963, CRA038005, CRA038059) that are publicly accessible at https://ngdc.cncb.ac.cn/gsa.
