# Supplementary material for: Comprehensive methylome and transcriptome profiling reveals specific biomarkers for bovine viral diarrhea virus persistent infection in calves
Source: Front Immunol. 2026 Mar 10;17:1763258. doi: 10.3389/fimmu.2026.1763258 (PMC13008631; doi:10.3389/fimmu.2026.1763258)
Supplement: Supplementary file 2 [file Table1.docx]

Supplementary Material

# Supplementary Figures


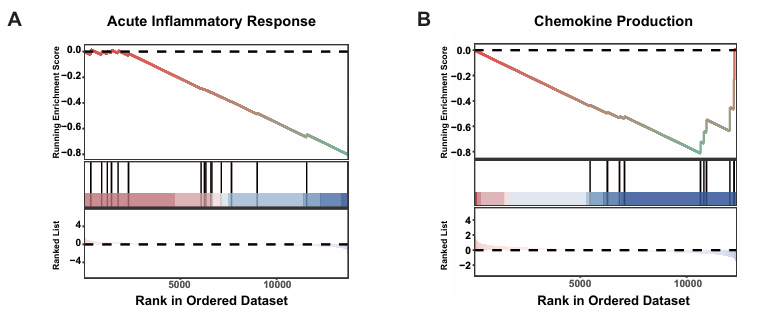


**Supplementary Figure 1.** **GSEA significantly downregulated pathways.** **A)** Significantly downregulated pathways in PI calves relative to normal calves (adjusted *p* < 0.05). **B)** Significantly downregulated pathways in trojan dams relative to normal dams (adjusted *p* < 0.05).


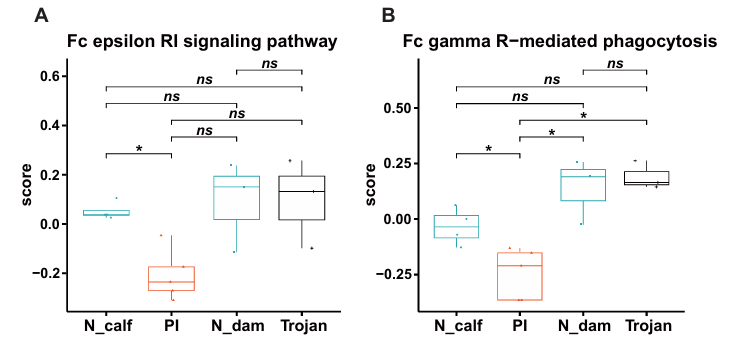


**Supplementary Figure 2.** **GSVA scores of immune-related pathways in each group.** * indicates *p* < 0.05; ns indicates *p* > 0.05 N_calf: normal calf group; PI: persistently infected (PI) calf group; N_dam: Dam group of normal calves; Trojan: Dam group of PI calves.

**
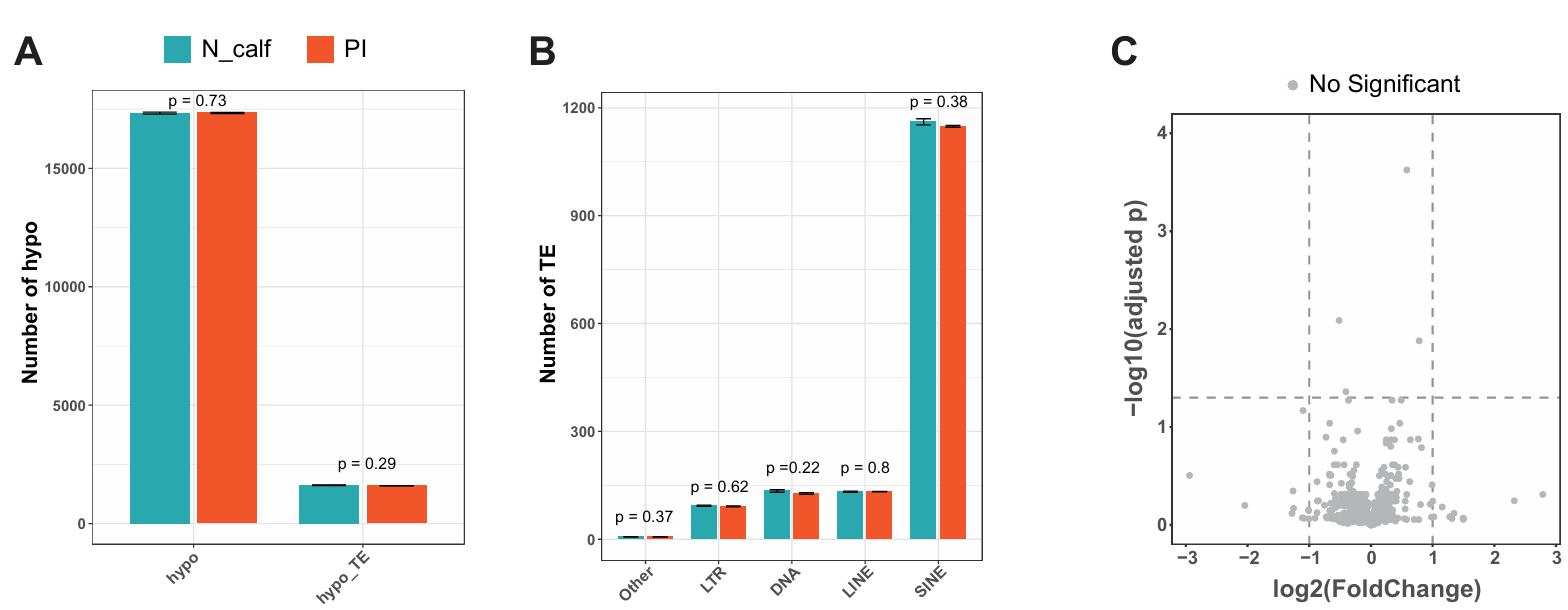
**

**Supplementary Figure 3. Transposable element (TE) features in calves. A)** Number of HMRs and HMRs overlapping with TEs in the normal calf and PI calf groups. **B)** Number of different types of TEs located in HMRs. **C)** Volcano plot of differential expression analysis of TEs in PI calf and normal calf groups.

**Supplementary Table**

**Supplementary Table** 1**: Pedigree correction.** Sheet IBD: The identity by descent (IBD) between mother and daughter was calculated using Plink; Sheet pedigree right: Identifying correct mother-daughter pairs for pedigree inference using EasyPC; Sheet pedigree error: Identifying incorrect mother-daughter pairs for pedigree inference using EasyPC.

**Supplementary Table 2: Transcriptome differential expression analysis.** Sheet calf_group: Results from the differential expression analysis comparing the PI calf group versus the normal calf group; Sheet dam_group: Results from the differential expression analysis comparing the Trojan dam group versus the normal dam group.

**Supplementary Table 3:** **Enrichment analysis of significantly differentially expressed genes in the calf groups.** Sheet GO_upregulated_gene: Significantly enriched GO pathways among the upregulated genes in calf groups (*p* < 0.05); Sheet GO_downregulated_gene: Significantly enriched GO pathways among the downregulated genes in calf groups (*p* < 0.05); Sheet GSEA: Significantly enriched pathways from Gene Set Enrichment Analysis (GSEA) in the calf groups (adjusted *p* < 0.05).

**Supplementary Table 4:** **Enrichment analysis of significantly differentially expressed genes in the dam groups.** Sheet GO_upregulated_gene: Significantly enriched GO pathways among the upregulated genes in dam groups (*p* < 0.05); Sheet GO_downregulated_gene: Significantly enriched GO pathways among the downregulated genes in dam groups (*p* < 0.05); Sheet GSEA: Significantly enriched pathways from Gene Set Enrichment Analysis (GSEA) in the dam groups (adjusted *p* < 0.05).

**Supplementary Table 5:** **Transcription factor scan of DMRs.** Sheet motif results of calf groups: Transcription factor binding motifs detected in differentially methylated regions (DMRs) of calf groups.; Sheet motif results of damgroups: Transcription factor binding motifs detected in differentially methylated regions (DMRs) of dam groups.

**Supplementary Table 6: RIN value of samples.**
